# Spatial transcriptomics of *Ciona* adult brains reveals functional zonalization and insights into neural gland function

**DOI:** 10.1016/j.isci.2026.117346

**Published:** 2026-08-24

**Authors:** Xin Zeng, Fuki Gyoja, Ayana Maruo, Nanako Okawa, Ken-ichi Mizutani, Yutaka Suzuki, Kenta Nakai, Takehiro G. Kusakabe

**Affiliations:** 1 Human Genome Center, The Institute of Medical Science, The University of Tokyo, Tokyo 108-8639, Japan; 2 Department of Computational Biology and Medical Sciences, The University of Tokyo, Kashiwa 277-8563, Japan; 3 Institute for Integrative Neurobiology and Department of Biology, Konan University, Kobe 658-8501, Japan; 4 Graduate School of Pharmaceutical Sciences, Kobe Gakuin University, Kobe 650-8586, Japan

**Keywords:** *Ciona*, invertebrate chordate, ascidian, central nervous system, spatial transcriptomics, chordate evolution, super-resolved gene expression map

## Abstract

The ascidian *Ciona* is a pivotal chordate model for illuminating the evolutionary origins of the vertebrate brain. Here, spatial transcriptomics of the adult *Ciona* neural complex, combined with image-based computational super-resolution mapping, resolved distinct tissue domains including the cerebral ganglion, neural gland, ciliated funnel, neural gland duct/dorsal strand, and body wall muscle. Within the cerebral ganglion, high-resolution mapping revealed clear molecular zonalization separating the cortex and medulla, alongside regional specialization within the cortex itself. The neural gland exhibited localized enrichment of genes associated with extracellular matrix and cell-cell interactions. These spatial features suggest that the neural gland functions as a homeostatic and signaling interface, reminiscent of primitive vertebrate meninges or choroid plexus. Overall, this spatially defined gene expression map provides a foundational framework for understanding functional regionalization in the tunicate brain and its evolutionary relationship to vertebrate nervous systems.

## Highlights

• Spatially resolved transcriptomic atlas of the adult *Ciona* neural complex
• Super-resolution gene expression maps for ∼980 genes in the neural complex
• New marker genes for the neural gland and the cerebral ganglion subregions
• Similarity of the neural gland to the meninges and choroid plexus of vertebrates

## Graphical abstract

**Figure:**
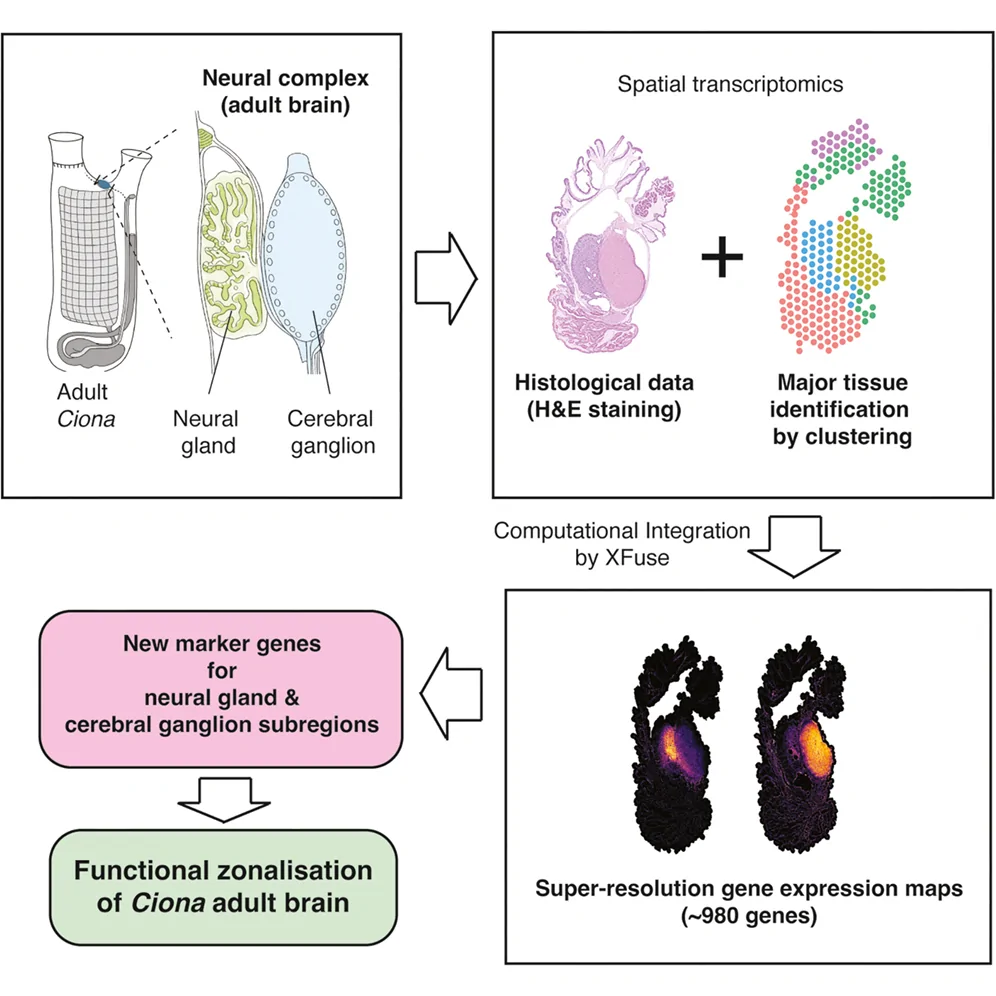

## Teaser

Neuroscience; Neuroanatomy; Transcriptomics

## Introduction

The central nervous system (CNS) of ascidians, the closest invertebrate relatives of vertebrates, provides an important model for understanding the evolution of the vertebrate brain.^1^ For instance, a recent comparative study between ascidian larval brain and mouse hypothalamus has provided insights into the evolutionary origin of vertebrate hypothalamic neuroendocrine systems.^2^ Although the larval nervous system of *Ciona* species (*C. intestinalis* and *C. robusta*) has been extensively characterized at the transcriptomic and cellular levels,^1^^,^^3^^,^^4^^,^^5^^,^^6^^,^^7^ the adult brain, which is referred to as the neural complex, has received comparatively less attention. One reason for this is technical. While it is relatively easy to analyze gene expression and function at the single-cell level in the brain of ascidian larvae using transient transgenic techniques or whole-mount methods, it is difficult to apply transgenic techniques or perform whole-mount analysis in the brain of adult ascidians, which take several weeks to reach maturity after metamorphosis.

The *Ciona* neural complex consists primarily of the cerebral ganglion and the neural gland and is thought to perform integrative and neuroendocrine-like functions.^8^ Cell lineage tracing studies have revealed that the cerebral ganglion develops during metamorphosis from the neural progenitor cells in the larval brain.^9^^,^^10^ However, processes and mechanisms of the neural complex development are still largely unknown. This is primarily due to a lack of information regarding the types of cells that constitute the neural complex and their distribution. Recent anatomical mapping studies have described the peripheral innervation architecture of the adult nervous system,^11^ and neuropeptidomic imaging has revealed regional neurochemical organization within the cerebral ganglion.^12^ However, a spatially resolved transcriptomic map linking cellular identity, molecular programs, and anatomical structure across the adult neural complex is still lacking. Recent advances in spatial transcriptomics enable genome-wide measurement of gene expression while preserving tissue architecture.^13^ In addition, computational approaches that integrate histological image features with transcriptomic measurements can further refine spatial boundaries and reveal substructures beyond the native resolution.^14^ These advances provide an opportunity to systematically characterize the spatial molecular architecture of the neural complex.

Here, we generated spatial transcriptomic data for the adult neural complex of *C. robusta* (also called *C. intestinalis* type A) and applied computational methods to improve spatial resolution. The resulting high-resolution spatial maps enabled systematic identification of spatially restricted marker genes across the neural complex. We further identified marker genes for the neural gland and three subregions of the cerebral ganglion.

## Results

### Spatial transcriptomic landscape of the adult brain of *Ciona*

**Figure 1:**
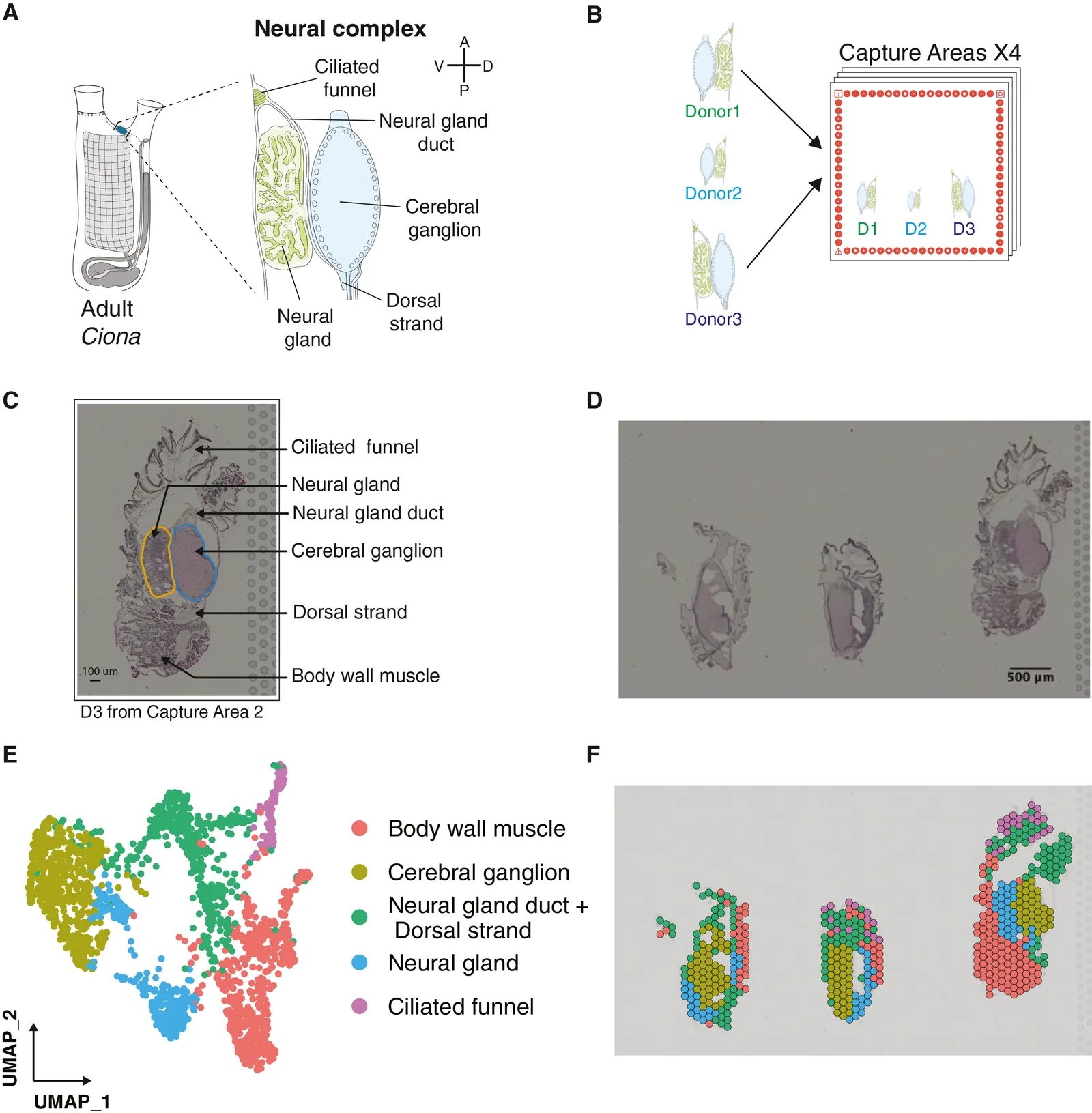
Spatial transcriptomic landscape of the adult *Ciona* brain (A) Schematic illustration of the adult *Ciona* neural complex (adapted from Osugi et al.^11^ and Olsson^15^), highlighting the two main brain structures, the cerebral ganglion and the neural gland. Orientation is indicated by anterior-posterior (A-P) and dorsal-ventral (D-V) axes. (B) Experimental design for spatial transcriptomics. Brain tissues from three adult individuals were sectioned serially, with four sections per individual distributed across the capture areas of a Visium slide. (C) Representative histological image from the Visium workflow showing anatomical identification of the cerebral ganglion and neural gland. The example shown is donor 3, a section from capture area 2. Scale bar: 100 μm. (D) Representative histological image including three sections in a capture area. The example shown is capture area 2. Scale bar: 500 μm. (E) UMAP plots of all transcriptomic spots colored by annotated tissues. (F) Spatial mapping of clusters onto the tissue sections shown in (D). Data were generated from three biologically independent adult *C. robusta* individuals (12 sagittal sections in total).

The adult *Ciona* brain is located in the anterior region of the animal and is mainly composed of two major structures: the cerebral ganglion and the neural gland (Figure 1A). We collected brain sections from three adult *Ciona* individuals using a balanced spatial replicate design (Figure 1B). For each individual, four serial sections from a comparable anatomical region were obtained and distributed across four capture areas of a Visium slide (Figures 1D and S1). Histological images acquired during the Visium workflow allowed clear identification of major anatomical features within the brain sections, including the neural gland and cerebral ganglion (Figure 1C). Spatial gene expression profiling of these sections was performed using the 10× Genomics Visium platform. The resulting Visium libraries produced approximately 3.9 × 10^8^ to 4.4 × 10^8^ reads per sample, with 414–532 spots detected under tissue. Across samples, the median UMI counts per spot ranged from ∼12,000 to ∼20,000, and a median of ∼3,300–4,200 genes were detected per spot (Figure S2A). In total, 1,949 spots were retained for downstream analyses.

We performed downstream analyses using Seurat v4.^16^ To reduce technical variation across samples and individuals, we applied canonical correlation analysis (CCA) to integrate Visium datasets prior to clustering. After batch correction, spots from different samples and individuals were well mixed in the integrated low-dimensional space, indicating effective removal of batch effects (Figures S2B and S2C). We then performed graph-based community detection on the integrated data and identified five clusters at the selected resolution (Figure 1E; see STAR Methods). Similar clustering patterns were reproducibly observed across individuals and Visium slides, indicating that the identified clusters were not driven by individual-specific effects (Figures 1F and S3).

### Tissue annotation for the adult brain of *ciona*

**Figure 2:**
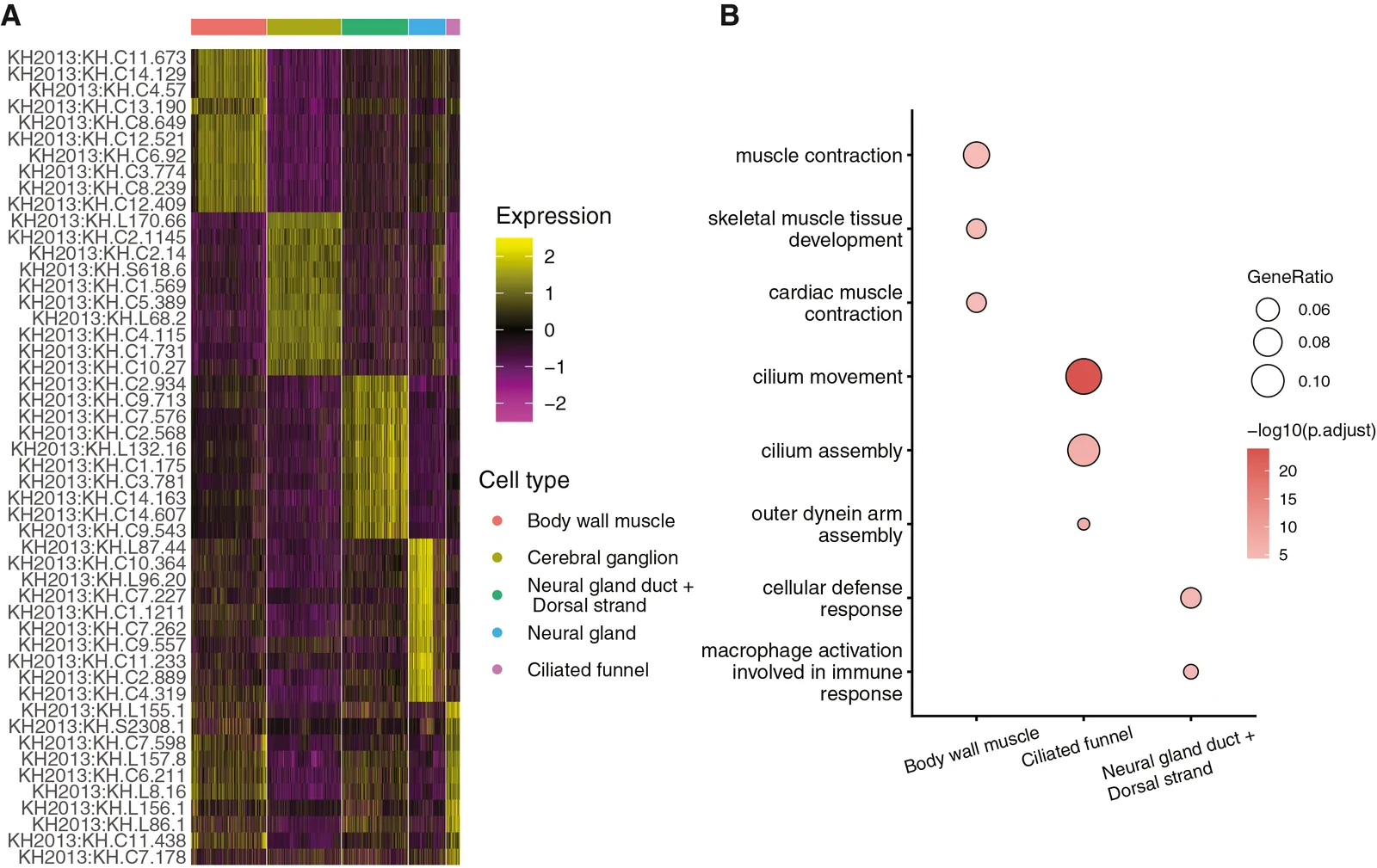
Annotation of clusters in the adult brain of *Ciona* (A) Heatmap showing scaled expression levels of representative marker genes across all tissue types. (B) Gene Ontology (GO) enrichment analysis of marker genes for body wall muscle, ciliated funnel, and neural gland duct/dorsal strand. Analyses were performed using all 1,949 quality-controlled Visium spots derived from three biologically independent adult animals.

We assigned each cluster to anatomical structures in the adult *Ciona* brain by combining histological observations and differentially expressed genes. Specifically, we identified marker genes for each spatial cluster based on differential expression analysis (see STAR Methods), which showed clear cluster-specific expression patterns (Figure 2A). Clusters corresponding to the cerebral ganglion and neural gland were identified based on their spatial correspondence to these structures in histological images across sections. For clusters whose anatomical identity could not be unambiguously determined from histology alone, we relied on complementary lines of evidence. We annotated body wall muscle based on strong enrichment of Gene Ontology (GO) terms related to muscle structure and contraction among its marker genes (Figure 2B). In addition, we annotated the ciliated funnel by its anterior-most position and the enrichment of GO terms associated with ciliary organization and motile cilia. Furthermore, we annotated the neural gland duct together with the dorsal strand, based on its reproducible spatial positioning relative to the cerebral ganglion and neural gland across individuals and Visium slides.

### Construction of super-resolution gene maps of the *Ciona* brain

**Figure 3:**
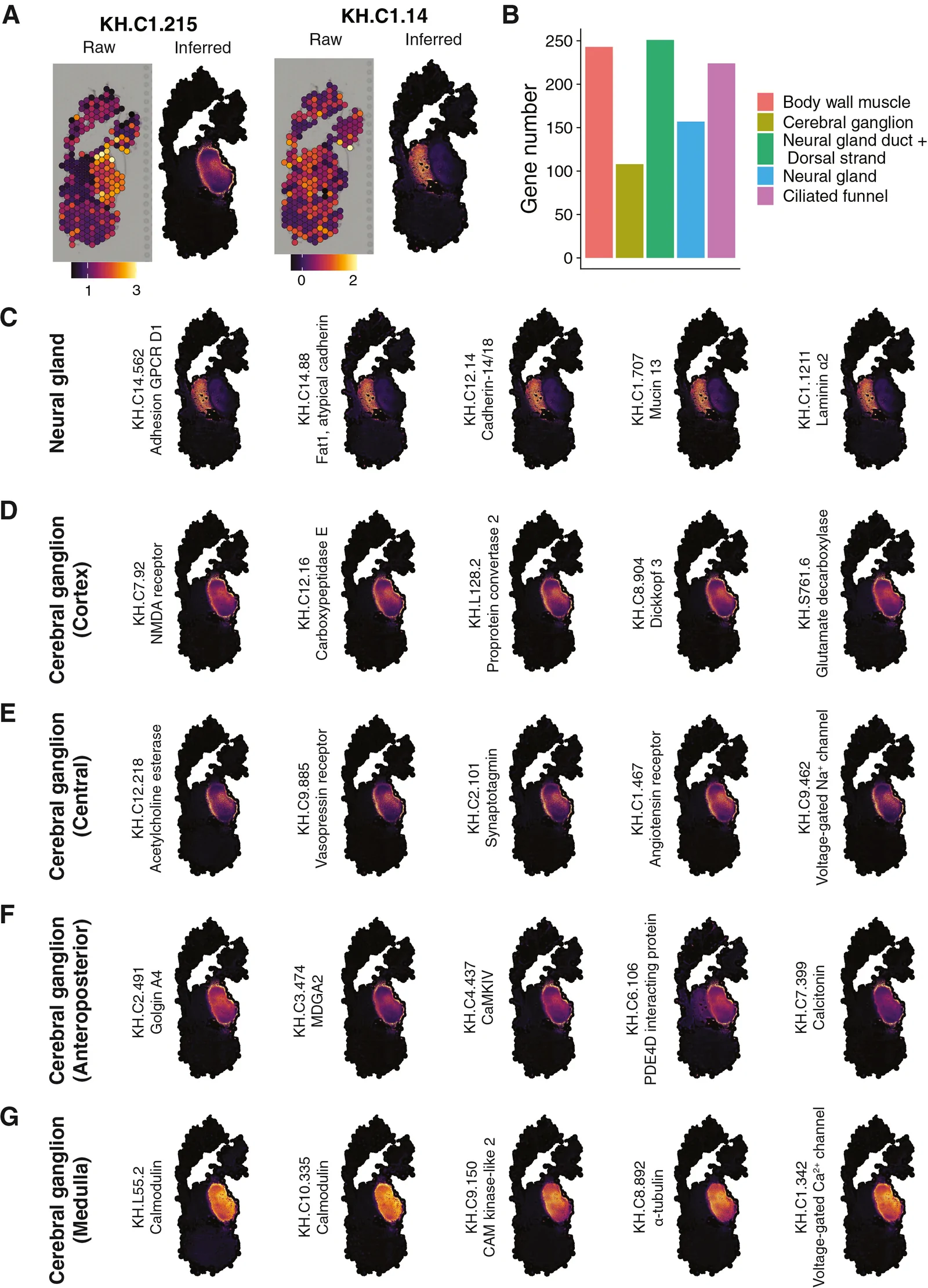
Super-resolution spatial gene maps reveal region-specific marker genes in the adult *Ciona* neural complex (A) Representative examples of raw Visium spot-level expression maps and corresponding Xfuse-inferred super-resolution maps for selected genes. (B) Number of genes generated by Xfuse for each major anatomical cluster. (C–G) Super-resolution expression maps of representative marker genes enriched in the neural gland (C) and different regions of the neural gland: pan-cortex (D); central cortex (E); anteroposterior cortex (F); and medulla (G). Representative examples are shown from donor 3, capture area 2, and are representative of the spatial expression patterns observed across all three biological replicates.

Although the spatial clusters captured major anatomical structures of the *Ciona* brain, the resolution of Visium transcriptomics data alone limited our ability to resolve finer substructures. To overcome this limitation, we integrated transcriptomic information with spatial image features derived from histological sections using Xfuse.^14^ Compared with raw Visium spot-level expression maps, Xfuse-inferred gene expression maps displayed sharper spatial boundaries and more refined internal organization, enabling clearer delineation of anatomical domains (Figure 3A). In addition, these refined spatial patterns were independently supported by previously published *in situ* hybridization data,^8^^,^^17^ in which the localization of *ci-galp* (KH.S618.6), *ci-ntlp-A* (KH.C2.14), *ci-vfyl/l* (KH.C2.1145), *ci-lf* (KH.C10.27), and *vasopressin receptor* (KH.C9.885) signals consistently showed enrichment in the cortical region of the cerebral ganglion and that of *somatostatin receptor* (KH.C8.337) confined to the neural gland, matching the domains identified by spatial transcriptomics (Figure S4). Although the *in situ* hybridization signal for the *vasopressin receptor* gene was also detected in the neural gland,^17^ the expression level was much lower in the neural gland than in the cerebral ganglion as shown in the raw spatial gene expression map (Figure S4C). We then systematically generated super-resolution gene expression maps for all cell-type-specific differentially expressed genes using Xfuse, resulting in approximately 980 high-resolution expression maps in total (Figure 3B).

### Identification of new marker genes for the neural gland

Using super-resolution gene expression maps, we identified a set of new marker genes with spatially restricted expression patterns for the neural complex. In the neural gland, 85 genes showed highly consistent and confined enrichment within this region across individuals (Figure 3C; Table S1). The list of these genes illuminates distinct physiological roles of this organ. A number of genes are related to extracellular matrix (ECM), including KH.C1.1211 (laminin), KH.C5.562 (dystroglycan), KH.C8.476 (lysyl oxidase like 3), KH.C9.371 (discoidin domain receptor tyrosine kinase 2), KH.C13.31 (secreted protein acidic and cysteine rich), KH.L87.44 (versican), KH.L139.15 (hemicentin-1), KH.C13.129 (sushi, von Willebrand factor type A, EGF, and pentraxin domain containing 1), KH.C1.707 (mucin 13), and KH.C1.1291 (matrix metallopeptidase 16). Another conspicuous feature is the expression of prospective neural support and axon guidance molecules, such as KH.C12.72 (netrin 1), KH.C5.524 (netrin 4), KH.C5.490 (netrin receptor unc-5), KH.L18.70 (neuregulin 2), KH.C11.251 (nerve growth factor receptor), and KH.S749.1 (glial fibrillary acidic protein, GFAP). Transmembrane receptors and adhesion molecules are also noticeable, including KH.C11.90/KH.C8.337 (somatostatin receptors), KH.C2.341 (G-protein-coupled receptor 107), KH.C14.202 (insulin receptor-related receptor), KH.C9.149 (prostaglandin I2 receptor), KH.C9.260 (frizzled class receptor 5/8), KH.C14.562 (adhesion GPCR D1), KH.C14.88 (FAT atypical cadherin 1), and KH.C12.14 (cadherin 18). The marker genes for the neural gland also include genes for solute carrier family (SLC) transporters (KH.C7.199, KH.L4.19, and KH.S215.18), calponin (KH.C4.559), and proteins involved in cell growth and differentiation, such as KH.C2.605 (hedgehog interacting protein), KH.C2.957 (msh homeobox), KH.C4.236 (Elf3/Ehf transcription factor), KH.C7.22 (ectodysplasin A), and KH.L112.16 (Jun/AP-1 transcription factor subunit). The coordinated enrichment of these molecules suggests distinct cell-cell interaction and extracellular environment properties within the neural gland, consistent with its proposed secretory and interface-related roles.

### Identification of new marker genes for the cerebral ganglion

We next focused on the cerebral ganglion. The cerebral ganglion is divided into the cortex and medulla.^18^^,^^19^ The super-resolution gene expression maps revealed that spatial expression patterns could be broadly classified into four major groups: (1) uniformly expressed in the cortex (cortex), (2) predominantly expressed in the central cortex region, particularly in the ventral region facing the neural gland (central), (3) predominantly expressed in anterior and posterior parts of the cortex (anteroposterior), and (4) predominantly expressed in the inner medulla region (medulla). For the cortex (Figure 3D; Table S2), we identified marker genes including KH.C7.92 (NMDA receptor), KH.C12.16 (carboxypeptidase E), KH.L128.2 (proprotein convertase 2), KH.S761.6 (glutamate decarboxylase), KH.C6.11 (aminopeptidase O), KH.S618.6 (galanin-like neuropeptide precursor), KH.C8.904 (Dickkopf 3), and KH.C1.215 (acid-sensing ion channel subunit 1/2/3). Many of these genes are associated with the biogenesis of gamma-aminobutyric acid (GABA) and neuropeptides, and synaptic function and proton-gated ion channel activity, suggesting active neuronal signaling and peptide processing activities in this region. For the central cortex domain (Figure 3E; Table S3), we identified marker genes including KH.C1.421 (4-aminobutyrate aminotransferase, ABAT), KH.C1.498 (choline *O*-acetyltransferase), KH.C12.218 (acetylcholine esterase), KH.C9.885 (vasopressin receptor), KH.C1.1067 (neuronal calcium sensor 1), KH.C2.101 (synaptotagmin), KH.C5.389 (tachykinin precursor), KH.L22.28 (type B GABA receptor), KH.C1.467 (μ-opioid/somatostatin/angiotensin receptor-like), KH.S1051.1/KH.S1104.4 (gonadotropin-releasing hormone, GnRH, precursor), and KH.C9.462 (voltage-gated Na^+^ channel). These genes suggest specialized neurotransmitter response and synaptic vesicle release properties in this subregion. For the anteroposterior distal regions (Figure 3F; Table S4), we identified marker genes including KH.C2.491 (golgin A4), KH.C4.437 (Ca^2+^/calmodulin-dependent protein kinase IV), KH.C6.106 (phosphodiesterase 4D interacting protein), KH.C3.474 (MAM domain containing glycosylphosphatidylinositol anchor 2; MDGA2), and KH.C7.399 (calcitonin). For the medulla (Figure 3G; Table S5), we identified marker genes including KH.L55.2 and KH.C10.335 (calmodulin family members), KH.C9.150 (doublecortin-like and CAM kinase-like 2), KH.C8.892 (α-tubulin), KH.L116.85 (β-tubulin), and KH.C1.342 (voltage-gated Ca^2+^ channel). These genes are related to calcium signaling and cytoskeletal structure, consistent with structural core and regulatory function in the cerebral ganglion.

## Discussion

In this study, we present a spatially resolved transcriptomic atlas of the adult *Ciona* neural complex. Clustering-based analysis defined the major tissue domains of the adult neural complex, providing a comprehensive transcriptional framework for understanding its organization and surrounding structures. In addition to the cerebral ganglion and neural gland, the atlas also resolves adjacent tissues such as the ciliated funnel, the neural gland duct, and the dorsal strand, offering a spatial molecular resource that may facilitate future studies of these structures.

To further refine spatial resolution in adult tissues, we generated super-resolution gene expression maps by integrating histological image features with spatial transcriptomic data. This approach provides a scalable molecular reference that reduces reliance on gene-by-gene anatomical screening. Based on the super-resolution gene expression maps, we identified spatially restricted marker genes for both the neural gland and the cerebral ganglion. The neural gland displays a distinct molecular signature consistent with specialized structural and secretory functions, while the cerebral ganglion exhibits clear internal zonation, indicating functional compartmentalization within this compact brain. These findings suggest that the spatial organization of neuronal and neuroendocrine programs is already established in a simple chordate nervous system. Previous studies have identified conserved molecular signatures shared between *Ciona* and vertebrates in particular cell types, including photoreceptor-related cells^20^ and hypothalamic neuroendocrine lineages.^2^ The atlas presented here extends these efforts by providing a comprehensive spatial framework for comparing conserved molecular signatures across species, thereby enabling systematic investigation of how regional specialization of the vertebrate brain emerged during evolution.

Special attention has been paid to the neural gland due to its distinct anatomical features and possible homology to vertebrate organs. The proposed homologous organs in vertebrates include adenophypophysis^21^^,^^22^ and choroid plexus.^17^ Various functions have been proposed for the neural gland, including an endocrine organ,^22^^,^^23^^,^^24^ an excretory organ,^25^ an exocrine organ,^17^ a lymph-producing organ,^23^ an osomoregulatory organ,^17^^,^^26^ and an organ that mediates regeneration of the cerebral ganglion.^27^^,^^28^ Despite these interests and studies, the physiological and developmental roles of this organ remain elusive. Accordingly, only a few genes have been identified as specifically expressed in the neural gland.^8^^,^^17^

In the present study, we identified 85 neural gland-specific genes. These genes support some of the previously proposed functions and homology of the neural gland. Expression of various SLC transporters with calponin suggests its role in regulating chemical (ions, amino acids, and peptides) and physical (pressure or flow of fluid) environment around the neural complex, or acting as a selective barrier. A osmoregulatory role is also suggested by the expression of various GPCRs for the putative osmoregulatory neuropeptides; some of these receptors are also expressed in the vertebrate choroid plexus.^17^ Deyts et al. proposed that the neural gland is an osmoregulatory organ and might be homologous to the choroid plexus of vertebrates. Furthermore, expression of GFAP and neural guidance cues (netrins, netrin receptors, and neuregulin 2) supports a proposed role of the neural gland in development and regeneration of the cerebral ganglion. Our results are compatible with this idea and further extend its neuroprotective and supportive roles. The richness of ECM proteins and putative protective and instructive roles for the brain are also reminiscent of the meninges of vertebrates, especially the pia mater, where the choroid plexus derive from. Thus, the neural gland may represent a primitive form of the meninges-choroid plexus system in vertebrates.

Previously, the cortex and medulla were recognized as distinct regions in the cerebral ganglion.^18^^,^^19^ Our super-resolved gene maps identified four groups of gene expression patterns in the cerebral ganglion: (1) uniformly expressed in the cortex, (2) predominantly expressed in the central cortex region, particularly in the cortex region facing the neural gland, (3) predominantly expressed in the anterior and posterior parts of the cortex, and (4) predominantly expressed in the medulla. Pan-cortex and central cortex genes include genes for the biogenesis of neurotransmitters and neuropeptides, their receptors, and various proteins involved in synaptic function, suggesting that the cortex contains a number of neurons and serves as the primary site of neuronal activity. On the contrary, the anteroposterior distal regions of the cortex show a distinct molecular signature in which proteins associated with neuronal and synaptic functions are less prominent, suggesting a distinct molecular function of these regions. In the medulla, genes for proteins involved in intracellular Ca^2+^ signaling are conspicuously expressed together with microtubule components. Expression of these genes suggests the medulla serves as the structural core of the cerebral ganglion and plays a regulatory role with active Ca^2+^ signaling. In summary, the present analysis revealed previously unrecognized molecular heterogeneity in the cerebral ganglion, especially within the cortex.

### Limitations of the study

Several limitations should be acknowledged. First, the current spatial maps are derived from spot-based measurements, and integration with single-cell RNA sequencing data from the adult brain will be necessary to further refine cell-type–specific expression patterns. Such integration would enable more precise delineation of cellular heterogeneity within each anatomical domain.^29^ Second, the XFuse-generated super-resolution map occasionally emphasizes regions that predominantly express the gene and omits regions with lower expression, as shown in Figure S3C. This current limitation also recalls the importance of integration with single-cell RNA sequencing data. Third, we did not perform extensive regulatory network analysis. In *Ciona*, widespread *trans*-splicing complicates transcript-level assignment and regulatory inference,^30^ posing challenges for conventional gene regulatory network reconstruction. Future studies combining improved transcript annotation, single-cell data, and perturbation experiments will be essential for dissecting regulatory mechanisms underlying the spatial programs described here. Fourth, the proposed homology of the neural gland to the meninges-choroid plexus system in vertebrates is hypothetical and speculative. Future functional experiments, such as gene knockouts and/or physiological assays, will be important to evaluate the evolutionary hypothesis.

## Resource availability

### Lead contact

Further information and requests for resources should be directed to and will be fulfilled by the lead contact, Takehiro G. Kusakabe (tgk@konan-u.ac.jp).

### Materials availability

This study did not generate new unique reagents.

### Data and code availability

• FASTQ sequencing data generated in this study are publicly available in the Gene Expression Omnibus (GEO) under accession number GSE327796. Processed data are publicly available on figshare (https://doi.org/10.6084/m9.figshare.31915545).
• All custom codes generated for this study are publicly available on GitHub at https://github.com/xzengComBio/Ciona_ST and figshare (DOI: https://doi.org/10.6084/m9.figshare.31915545).
• Any additional information required to reanalyze the data reported in this paper is available from the lead contact upon request

## Acknowledgments

We thank the National BioResource Project of MEXT and all members of the Maizuru Fisheries Research Station and Yutaka Satou Lab of Kyoto University and the Misaki Marine Biological Station and Manabu Yoshida Lab of the University of Tokyo for providing us with *C. intestinalis* type A adults. We also thank the Graduate School of Maritime Sciences, Kobe University, for generously allowing us to collect *C*. *intestinalis* type A on the campus. Computational resources were provided by the supercomputer system SHIROKANE at the Human Genome Center, Institute of Medical Science, the University of Tokyo. This research was supported by 10.13039/501100001691KAKENHI grants-in-aid for Scientific Research (23K27185, 23H02492, 22K06189, and 21K19280) from the 10.13039/501100001691Japan Society for the Promotion of Science; Hirao Taro Foundation of KONAN GAKUEN for Academic Research and the 10.13039/100007449Takeda Science Foundation (2015021209, in part). X.Z. was supported by 10.13039/501100001695JST 10.13039/501100025019SPRING (JPMJSP2108).

## Author contributions

Conceptualization, X.Z., F.G., T.G.K., and K.N.; Visium data acquisition, A.M., N.O., K.-i.M., T.G.K., and Y.S.; data curation, X.Z. and T.G.K.; formal analysis, X.Z.; investigation, X.Z., F.G., A.M., and T.G.K.; writing – original draft, X.Z., F.G., and T.G.K.; writing – review and editing, X.Z., F.G., T.G.K., and K.N.; funding acquisition, T.G.K., Y.S., and K.N.; resources, K.-i.M., Y.S., T.G.K., and K.N.; supervision, T.G.K. and K.N.

## Declaration of interests

The authors declare that they have no competing interests.

## STAR★Methods

### Key resources table

**Table:** 

REAGENT or RESOURCE	SOURCE	IDENTIFIER
**Biological samples**		

Adult *Ciona robusta* neural complex	National BioResource Project (Japan)	N/A

**Critical commercial assays**		

Visium Spatial Tissue Optimization Slide & Reagent Kit	10× Genomics	Cat#1000193
Visium Spatial Gene Expression Slide & Reagent Kit	10× Genomics	Cat#1000184

**Deposited data**		

Raw spatial transcriptomic sequencing data	This paper; GEO	GSE327796
Processed spatial transcriptomic data	This paper; figshare	https://doi.org/10.6084/m9.figshare.31915545
Analysis code	This paper; GitHub	Code: https://doi.org/10.6084/m9.figshare.31915545; Github: https://github.com/xzengComBio/Ciona_ST

**Software and algorithms**		

Space Ranger version 1.3.1	10× Genomics	https://www.10xgenomics.com/support/software/space-ranger
Seurat version 4	Stuart et al.^16^	https://satijalab.org/seurat
XFuse	Bergenstråhle et al.^14^	https://github.com/ludvb/xfuse
R version 4.4	R Foundation	https://www.r-project.org

### Experimental model and study participant details

#### Biological materials

Sexually mature adults (2–3 months old, hermaphrodites) of *Ciona robusta* (also called *Ciona intestinalis* type A) were provided by Satou’s lab, (Kyoto University), under the support of the National Bio Resource Project (NBRP) of the Ministry of Education, Culture, Sports, Science and Technology of Japan (MEXT). These *Ciona* individuals were of genetically monitored closed colonies reared in the Maizuru Fisheries Research Station of Kyoto University.^31^ These were maintained in indoor tanks of artificial seawater (ASW) (Marine Art BR; Tomita Pharmaceutical, Tokushima, Japan) at 18-19°C under constant light in the Kusakabe laboratory of Konan University for seven days until tissue sampling. The body length of the individuals used was approximately 8 to 10 cm long.

### Method details

#### Tissue preparation and VISIUM sequencing library construction

*Ciona* whole brains were surgically dissected from mature adult individuals, embedded in Tissue-Tek OCT compound (Sakura Finetek, Tokyo, Japan), and sectioned at 10-μm thickness using a cryostat (HM525, Microm). Tissue fixation and staining were as described in the Methanol Fixation, H&E Staining & Imaging for Visium Spatial Protocols (10X Genomics; #CG000160 Rev C). Tissue permeabilization optimization was performed using Visium Spatial Tissue Optimization Slide & Reagent Kit (10 × Genomics, #1000193) according to the Visium Spatial Tissue Optimization User Guide Rev B (10 × Genomics, #CG000238). The optimal tissue permeabilization time for *Ciona* brains was determined as 12 min. The optimized condition was then used for Visium Spatial Gene Expression library preparation. The four capture areas on the Visium Gene Expression Slide (#2,000,233) contained serial sections from an embedded brain block, including three brains from different individuals. When embedded, the whole brain and associated tissues were aligned in the same direction so that each capture area contained three sagittal sections of three whole brains. Each capture area of the Visium Gene Expression Slide contained approximately 5000 barcoded spots of 55 μm in diameter (100-μm center-to-center spacing between spots). The sequencing library was prepared using Visium Spatial Gene Expression Slide & Reagent Kit (10 × Genomics, #1000184) according to the Visium Spatial Gene Expression User Guide Rev C (10X Genomics, #CG000239) and sequenced on a NovaSeq 6000 system (Illumina). Sequencing was performed with the recommended protocol (read 1: 28 cycles; i7 index read: 10 cycles; i5 index read: 10 cycles; and read 2: 90 cycles), yielding between 391 million and 440 million sequenced reads.

#### Mapping and preprocessing

Sequencing output was processed through the Space Ranger 1.3.1 count function using default parameters. Reads were quantified using the KH2013 reference genome provided by the Ghost Database.^32^ The raw count matrices were subsequently imported into R and subjected to analysis using the Seurat 4 package.^16^ The data were log normalized and scaled by the SCTransform function,^33^ followed by batch effect correction using canonical correlation analysis. The corrected data were scaled for the principal component analysis (PCA). The corrected data was used for clustering based on a shared nearest-neighbor modularity optimization-based clustering algorithm with default parameters and resolutions at 0.2. Non-linear dimensionality reduction was conducted using UMAP.

We performed Gene Ontology enrichment analysis for cluster-specific marker genes identified from spatial transcriptomic data using a custom *Ciona* GO annotation derived from the Aniseed GAF file.^34^ Enrichment of Biological Process terms was tested with clusterProfiler,^35^ and the top-enriched GO terms for each cluster were visualized using dot plots.

#### Generation of super-resolution gene maps

We used xfuse^14^ to generate high resolution gene expression map for marker genes. First, the coordinates on each brain section were extracted from the original “tissue_positions_list.csv” file. Then, the transcriptomics and image information for each brain section were extracted using xfuse convert Visium function with a scale value setting to 0.2. Finally, the separated 12 transcriptomics and image section were trained by the xfuse model using the xfuse run function via Nvidia V100 GPU. The convergence of the model was achieved following a computational duration of approximately 7 hours. The four distinct patterns (cortex, central, anteroposterior, and medulla) of super-resolution gene maps were identified by manually inspecting all images for the cerebral ganglion-specific genes and then assigned each gene to one of these four patterns also by manual inspection (Tables S2–S5).

### Quantification and statistical analysis

Statistical analyses were performed in R (version 4.4). Cluster-specific marker genes were identified using the Wilcoxon rank-sum test implemented in Seurat. Gene Ontology enrichment analysis was performed using clusterProfiler with Benjamini–Hochberg correction for multiple testing. Unless otherwise specified, adjusted *p* values < 0.05 were considered statistically significant.
