# Supplementary material for: Spatial transcriptomics of Ciona adult brains reveals functional zonalization and insights into neural gland function
Source: iScience. 2026 Aug 24;29(9):117346. doi: 10.1016/j.isci.2026.117346 (PMC13542503; doi:10.1016/j.isci.2026.117346)
Supplement: Supplementary file 1 [file mmc1.pdf]

iScience, Volume *29*

Supplemental information

Spatial transcriptomics of *Ciona* adult brains reveals functional zonalization and insights into neural gland function

Xin Zeng, Fuki Gyoja, Ayana Maruo, Nanako Okawa, Ken-ichi Mizutani, Yutaka Suzuki, Kenta Nakai, and Takehiro G. Kusakabe

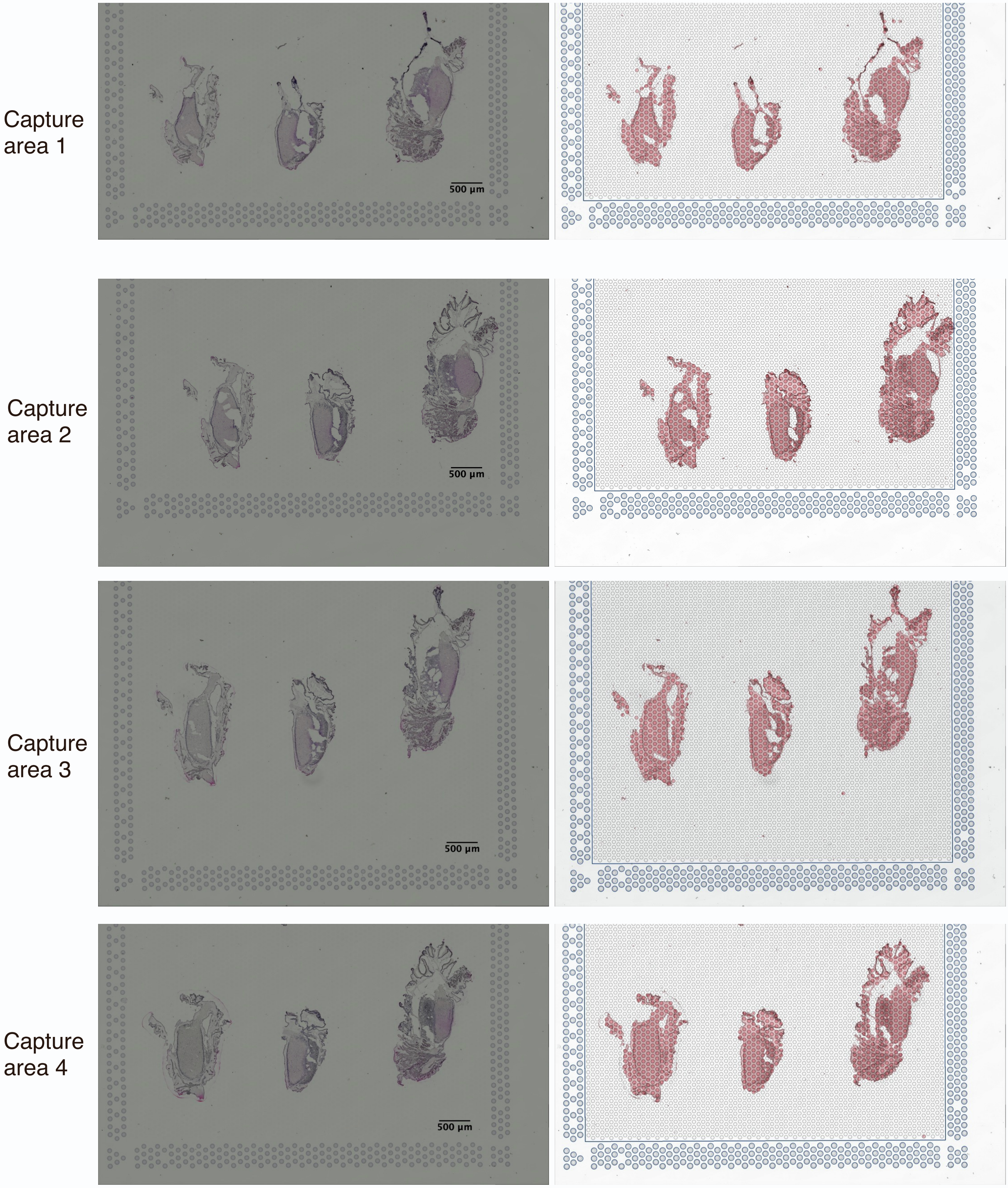

Supplemental Figure S1. Histological images and Visium spot alignments for all four sections used for spatial transcriptomic profiling.
For each section, the left panel shows the H&E-stained tissue image used for Visium spatial transcriptomics, and the right panel shows the corresponding Space Ranger tissue detection and spot alignment output. Scale bars, 500 µm.

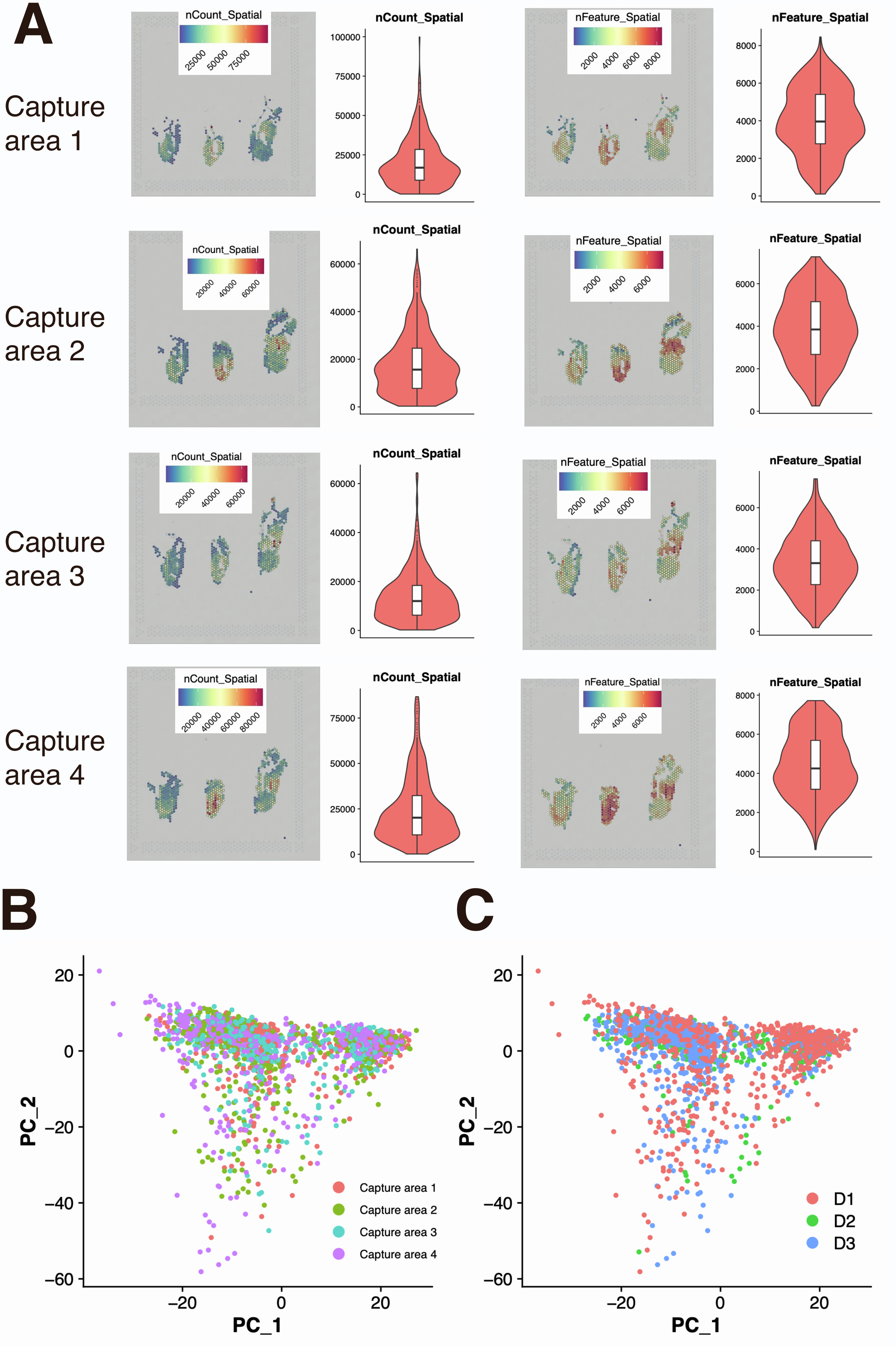

A
Capture area 1
Capture area 2
Capture area 3
Capture area 4
nCount_Spatial
nFeature_Spatial
B
C
PC_1
PC_2
Capture area 1
Capture area 2
Capture area 3
Capture area 4
D1
D2
D3

Supplemental Figure S2. Quality control and sample integration of Visium spatial transcriptomic datasets.

A Quality control metrics for four Visium slides. For each slide (Slide1–Slide4), spatial distributions of total UMI counts per spot (nCount_Spatial) and detected gene numbers per spot (nFeature_Spatial) are shown. Corresponding violin plots summarize the distribution of these metrics across all spots under tissue for each slide. **B-C** Principal component analysis (PCA) of all retained spots colored by slide identity (**B**) and biological donor (D1–D3) (**C**) after SCTransform normalization and CCA-based integration.

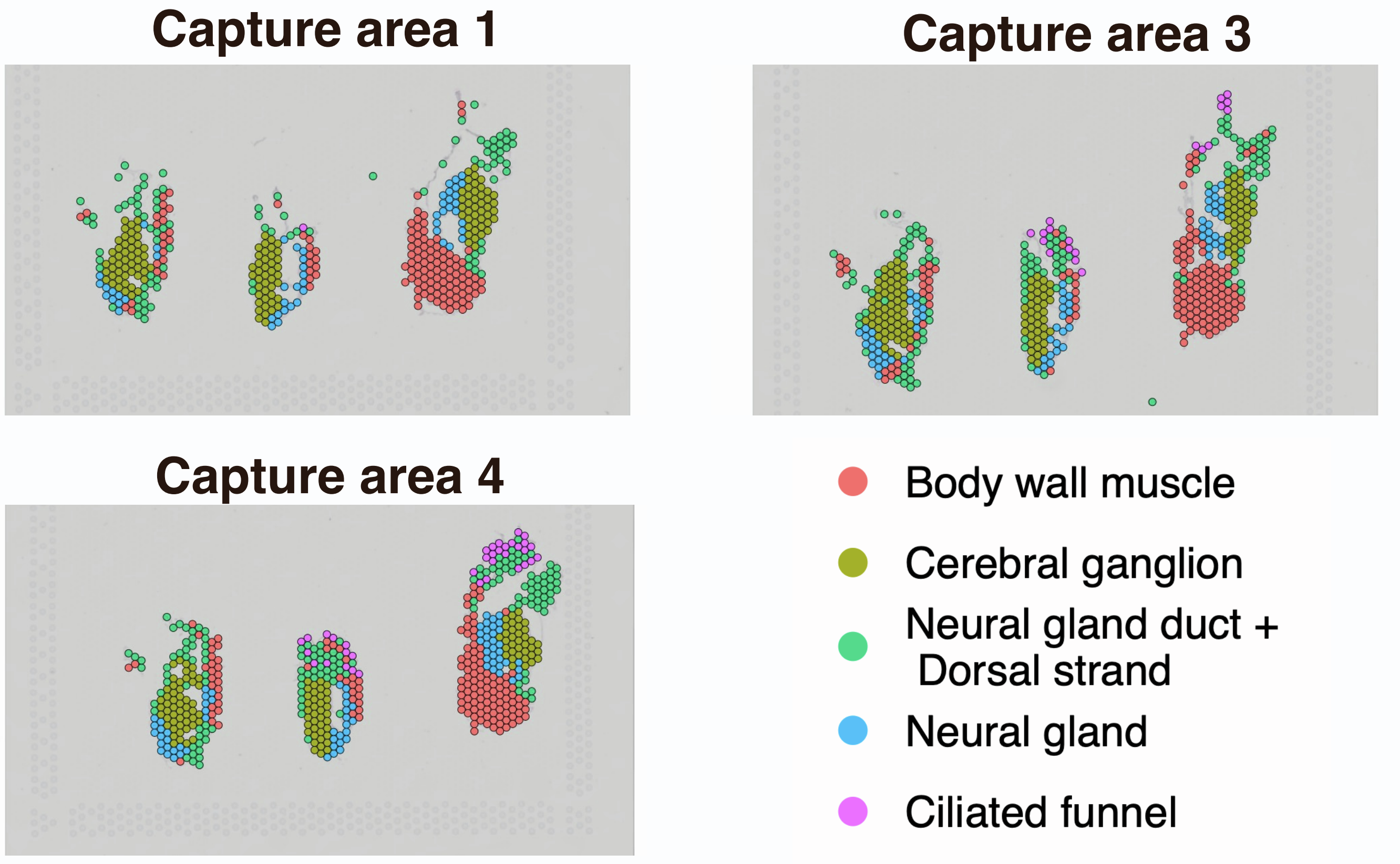

Supplemental Figure S3. Reproducibility of spatial clustering and functional annotation of identified tissue domains.
Spatial distribution of clusters across three capture areas (capture area 1, 2, and 3) of a Visium slide.

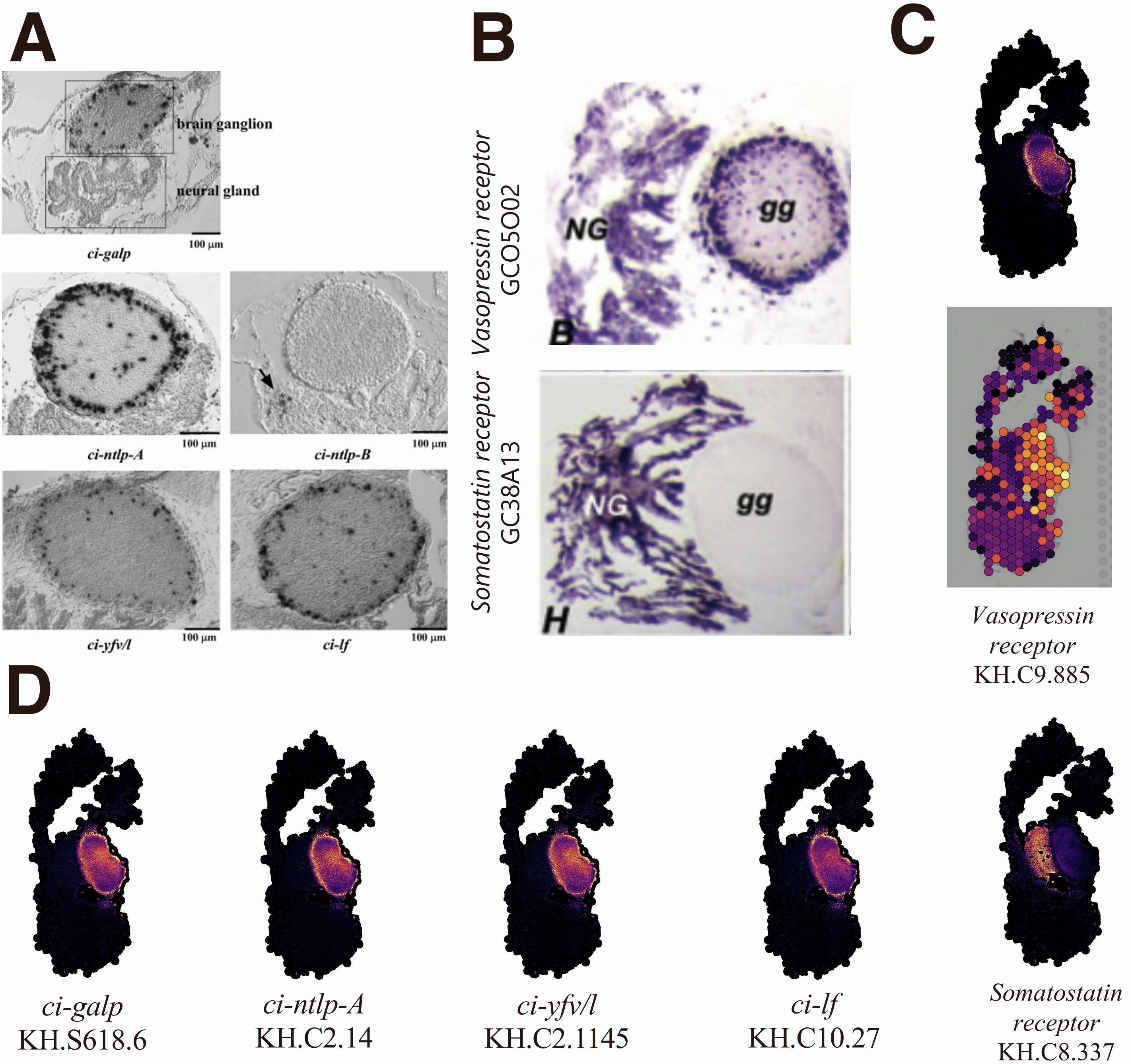

Supplemental Figure S4. Independent validation of spatial expression patterns by in situ hybridization.

A Representative in situ hybridization images showing expression patterns of *ci-galp*, *ci-ntlp-A*, *ci-ntlp-B*, *ci-vfyl/l*, and *ci-lf* in transverse sections of the adult neural complex (from Kawada et al. 2011). **B** Representative in situ hybridization images showing expression patterns of *vasopressin receptor* and *somatostatin receptor* (from Deyts et al. 2006) **C** Comparison of Visium spatial transcriptomic data and XFuse super-resolution reconstruction for vasopressin receptor. The upper panel shows the XFuse reconstruction, whereas the lower panel shows the original Visium spot-level expression. **D** XFuse super-resolution expression maps for marker genes corresponding to the *in situ* hybridization patterns shown in panel **A** and panel **B**.

Sapplemental Table S1. Neural gland-specific genes

Gene ID	Protein (blast best hit)	Biological function
**KH.C1.14**	ARF6 (ADP ribosylation factor 6)	Regulates endocytosis, membrane trafficking, and actin remodeling.
**KH.C1.205**	PRRT1B (proline rich transmembrane protein 1B)	Involved in synapse regulation and AMPA receptor trafficking.
**KH.C1.707**	MUC13 (mucin 13, cell surface associated)	Cell surface protection and signaling in epithelial layers.
**KH.C1.1211**	LAMA2 (laminin subunit alpha 2)	Basement membrane component; mediates cell adhesion and migration.
**KH.C1.1291**	MMP16 (matrix metallopeptidase 16)	Matrix degradation; critical for tissue remodeling and cell invasion.
**KH.C2.333**	CCN2 (cellular communication network factor 2)	Promotes cell adhesion, collagen synthesis, and ECM production.
**KH.C2.341**	GPR107 (G protein-coupled receptor 107)	G protein-coupled receptor involved in retrograde protein transport.
**KH.C2.399**	DGKQ (diacylglycerol kinase theta)	Converts diacylglycerol (DAG) to phosphatidic acid (PA) in signaling.
**KH.C2.422**	TES (testin LIM domain protein)	Scaffold protein involved in cell-matrix adhesion and cytoskeleton stability.
**KH.C2.605**	HHIP (hedgehog interacting protein)	Antagonist of the Hedgehog signaling pathway; regulates cell growth.
**KH.C2.889**	AICDA (activation induced cytidine deaminase)	Activation-induced cytidine deaminase; involved in DNA modification.
**KH.C2.957**	MSX2 (msh homeobox 2); Msx (msh homeobox)	Transcription factor governing tissue patterning and organogenesis.
**KH.C2.994**	RNF149 (ring finger protein 149)	E3 ubiquitin-protein ligase involved in protein degradation.
**KH.C3.37**	TAF1L (TATA-box binding protein associated factor 1 like)	Part of the transcription initiation complex for gene expression.
**KH.C3.149**	PLSCR1 (phospholipid scramblase 1)	Mediates phospholipid translocation (scrambling) and antiviral signaling.
**KH.C3.379**	ITIH3 (inter-alpha-trypsin inhibitor heavy chain 3)	Heavy chain of inter-alpha-trypsin inhibitor; stabilizes the ECM.
**KH.C4.140**	ARF3 (ADP ribosylation factor 3)	Regulates Golgi-to-plasma membrane vesicular trafficking.
**KH.C4.236**	EHF (ETS homologous factor); Elf3/Ehf	Transcription factor regulating epithelial cell differentiation.
**KH.C4.319**	TGM1 (transglutaminase 1)	Transglutaminase 1; catalyzes protein cross-linking for barrier formation.
**KH.C4.391**	FRMD6 (FERM domain containing 6)	Regulator of the Hippo signaling pathway (tumor suppression/growth).
**KH.C4.492**	PLEKHA5 (pleckstrin homology domain containing A5)	Scaffolding protein involved in intracellular signaling pathways.
**KH.C4.559**	CNN1 (calponin 1)	Regulates smooth muscle contraction by binding to actin.
**KH.C4.697**	ALDH1A2 (aldehyde dehydrogenase 1 family member A2)	Synthesizes Retinoic Acid; essential for developmental patterning.
**KH.C5.406**	not found	
**KH.C5.490**	UNC5C (unc-5 netrin receptor C)	Receptor for Netrin; guides axon growth and cell migration.
**KH.C5.524**	NTN4 (netrin 4)	Involved in basement membrane assembly and vascular stability.
**KH.C5.562**	DAG1 (dystroglycan 1)	Links the extracellular matrix (ECM) to the cytoskeleton.
**KH.C5.581**	TIMP2 (TIMP metallopeptidase inhibitor 2)	Inhibitor of matrix metalloproteinases; protects ECM from degradation.
**KH.C7.22**	EDA (ectodysplasin A)	Signaling molecule for the development of ectodermal tissues.
**KH.C7.199**	SLC23A1 (solute carrier family 23 member 1)	Sodium-dependent vitamin C (ascorbate) transporter.
**KH.C7.227**	TMPRSS15 (transmembrane serine protease 15)	Serine protease (Enterokinase) involved in protein processing.
**KH.C7.260**	TPM4 (tropomyosin 4)	Tropomyosin; regulates the actin cytoskeleton in non-muscle cells.
**KH.C7.262**	TFPI (tissue factor pathway inhibitor)	Inhibits the tissue factor-mediated blood coagulation cascade.
**KH.C8.337**	SSTR2 (somatostatin receptor 2)	GPCR for somatostatin; inhibits hormone secretion and cell growth.
**KH.C8.476**	LOXL3 (lysyl oxidase like 3)	Catalyzes the cross-linking of collagen and elastin in the ECM.
**KH.C9.149**	PTGIR (prostaglandin I2 receptor)	Receptor for prostacyclin; mediates vasodilation and anti-aggregation.
**KH.C9.260**	FZD5 (frizzled class receptor 5); Fzd5/8 (frizzled class receptor 5/8)	Wnt receptor; involved in cell polarity and developmental signaling.
**KH.C9.371**	DDR2 (discoidin domain receptor tyrosine kinase 2)	Tyrosine kinase receptor that binds collagen to regulate remodeling.
**KH.C9.398**	CCDC190 (coiled-coil domain containing 190)	Coiled-coil protein likely involved in ciliary or structural functions.

**KH.C9.615**	mannose receptor C1; lectin C-type domains	Mannose receptor; involved in endocytosis and immune recognition.
**KH.C9.616**	LMX1B (LIM homeobox transcription factor 1 beta); Lmx1	Transcription factor essential for limb and kidney development.
**KH.C9.859**	RAC1 (Rac family small GTPase 1)	Small GTPase regulating actin dynamics and cell motility.
**KH.C10.302**	CRNN (cornulin)	Cornulin; marker of squamous epithelial differentiation/stress response.
**KH.C10.364**	ATP8A2 (ATPase phospholipid transporting 8A2)	Phospholipid flippase critical for neural and retinal health.
**KH.C11.90**	SSTR2 (somatostatin receptor 2)	(See SSTR2 above) Second entry likely representing an isoform.
**KH.C11.233**	PVRIG (PVR related immunoglobulin domain containing)	Cell surface receptor involved in modulating immune responses.
**KH.C11.251**	NGFR (nerve growth factor receptor)	Nerve growth factor receptor; regulates neuronal survival and growth.
**KH.C11.325**	PHLDA3 (pleckstrin homology like domain family A member 3)	Pro-apoptotic protein; acts as a repressor of Akt signaling.
**KH.C11.577**	RGS4 (regulator of G protein signaling 4)	Regulates G protein signaling; highly expressed in the brain.
**KH.C11.605**	CD40LG (CD40 ligand)	Cytokine involved in immune signaling and inflammatory response.
**KH.C11.648**	SERPINB9 (serpin family B member 9)	Protease inhibitor; protects cells from granzyme-mediated apoptosis.
**KH.C11.667**	BTBD8 (BTB domain containing 8)	Involved in protein-protein interactions and signaling.
**KH.C12.14**	CDH18 (cadherin 18)	Neural cadherin involved in synaptic adhesion and connectivity.
**KH.C12.72**	NTN1 (netrin 1)	Chemotropic cue (Netrin-1) for axon guidance and migration.
**KH.C12.110**	DNAI7 (dynein axonemal intermediate chain 7)	Component of the dynein complex in motile cilia.
**KH.C12.394**	UBR2 (ubiquitin protein ligase E3 component n-recognin 2)	E3 ligase involved in the N-end rule protein degradation pathway.
**KH.C12.671**	FLNB (filamin B)	Cross-links actin filaments; essential for skeletal development.
**KH.C13.31**	SPARC (secreted protein acidic and cysteine rich)	Modulates cell-matrix interactions and matrix mineralization.
**KH.C13.39**	COCH (cochlin)	Component of the inner ear ECM; involved in structural integrity.
**KH.C13.129**	SVEP1 (sushi, von Willebrand factor type A, EGF and pentraxin doma	Large adhesion protein involved in tissue development and ECM.
**KH.C13.130**	MAP1LC3C (microtubule associated protein 1 light chain 3 gamma)	Plays a role in autophagy and cargo selection.
**KH.C13.178**	CRISPLD2 (cysteine rich secretory protein LCCL domain containing 2	Involved in lung development and ECM-related signaling.
**KH.C14.88**	FAT1 (FAT atypical cadherin 1)	Atypical cadherin regulating cell migration and Hippo signaling.
**KH.C14.188**	MINPP1 (multiple inositol-polyphosphate phosphatase 1)	Phosphatase that removes phosphate groups from inositol polyphosphates.
**KH.C14.202**	INSRR (insulin receptor related receptor)	Orphan receptor of the insulin receptor family; pH sensor.
**KH.C14.562**	ADGRD1 (adhesion G protein-coupled receptor D1)	Adhesion GPCR with roles in developmental signaling.
**KH.L4.19**	SLC36A2 (solute carrier family 36 member 2)	Proton-coupled amino acid transporter.
**KH.L18.70**	NRG2 (neuregulin 2)	Neuregulin-2; ligand for ErbB receptors in neural signaling.
**KH.L22.32**	CCDC200 (coiled-coil domain containing 200)	Coiled-coil protein with potential roles in structural organization.
**KH.L35.10**	SCN2B (sodium voltage-gated channel beta subunit 2)	Beta subunit of voltage-gated sodium channels; modulates excitability.
**KH.L35.11**	Receptor-type tyrosine-protein phosphatase epsilon precursor	Tyrosine phosphatase regulating signaling in bone and neural cells.
**KH.L41.66**	ERGIC3 (ERGIC and golgi 3)	Involved in protein transport between the ER and Golgi.
**KH.L71.1**	RNA binding protein with multiple splicing	RNA-binding protein found in retinal ganglion and smooth muscle cells.
**KH.L87.44**	VCAN (versican)	Versican; large proteoglycan essential for tissue hydration and migration.
**KH.L88.1**	XIAP (X-linked inhibitor of apoptosis)	Inhibitor of apoptosis; prevents programmed cell death.
**KH.L96.20**	TFPI (tissue factor pathway inhibitor)	Inhibitor of serine proteases in coagulation or tissue remodeling.
**KH.L112.16**	JUN (Jun proto-oncogene, AP-1 transcription factor subunit); Bziptun2	Transcription factor (AP-1) regulating stress response and growth.
**KH.L114.12**	not found	
**KH.L139.15**	HMCN1 (hemicentin 1)	Hemicentin-1; large ECM protein involved in tissue attachment.
**KH.L139.21**	ANKRD52 (ankyrin repeat domain 52)	Regulator of protein phosphatase 6 (PP6) activity.

**KH.S215.18**	SLC15A1 (solute carrier family 15 member 1)	Transporter for di- and tripeptides.
**KH.S391.6**	PNPLA7 (patatin like phospholipase domain containing 7)	Phospholipase involved in lipid metabolism.
**KH.S573.6**	Vascular endothelial growth factor C precursor	Growth factor for the development of lymphatic/vascular systems.
**KH.S749.1**	GFAP (glial fibrillary acidic protein)	Glial fibrillary acidic protein; marker for glial cell filaments.
**KH.S1035.3**	BRI3 (brain protein I3)	Brain protein I3; involved in lysosomal targeting and neural function.

Supplemental Table S2. Genes expressed uniformly in the cortex of the cerebral ganglion

Gene ID	Protein (blast best hit)	Biological function
**KH.C1.215**	ASIC1 (acid sensing ion channel subunit 1)	Proton-gated sodium channel; synaptic plasticity
**KH.C1.804**	IQGAP2 (IQ motif containing GTPase activating protein 2)	Regulation of the actin cytoskeleton and cell signaling
**KH.C1.943**	not found	
**KH.C2.14**	neurotensin (NT)-like peptide presursor; ci-ntlp-A	neurotensin (NT)-like peptides
**KH.C2.140**	TMSB10 (thymosin beta 10)	Actin-sequestering protein; cytoskeletal organization
**KH.C2.1145**	Tyr-Phe-Val/Leu (YFV/L) peptide family; ci-vfyl/l	novel peptide family in Ciona (Kawada et al., 2011)
**KH.C4.115**	HIP1 (huntingtin interacting protein 1)	Endocytosis and huntingtin-mediated signaling
**KH.C6.11**	AOPEP (aminopeptidase O (putative))	Proteolytic processing of bioactive peptides
**KH.C7.92**	GRIN2C (glutamate ionotropic receptor NMDA type subunit 2C)	NMDA receptor subunit; excitatory neurotransmission
**KH.C8.904**	DKK3 (dickkopf WNT signaling pathway inhibitor 3)	Modulation of the Wnt signaling pathway
**KH.C12.16**	CPE (carboxypeptidase E)	Processing of neuropeptides and peptide hormones
**KH.L24.12**	SLC22A2 (solute carrier family 22 member 2)	Organic cation transport across membranes
**KH.L128.2**	PCSK2 (proprotein convertase subtilisin/kexin type 2)	Neuropeptide maturation and hormone processing
**KH.S618.6**	Galanin-like neuropeptide precursor; ci-galp	Potential neuropeptide precursor (Galanin-like)
**KH.S761.6**	GAD1 (glutamate decarboxylase 1)	Synthesis of the inhibitory neurotransmitter GABA

Supplemental Table S3. Genes predominantly expressed in the central cortex region of the cerebral ganglion

Gene ID	Protein (blast best hit)	Biological function
**KH.C1.118**	SRRM4 (serine/arginine repetitive matrix 4)	Regulation of alternative splicing in neurons
**KH.C1.127**	LRRN2 (leucine rich repeat neuronal 2)	Cell-cell adhesion and neuronal signaling
**KH.C1.421**	ABAT (4-aminobutyrate aminotransferase)	Metabolism of the inhibitory neurotransmitter GABA
**KH.C1.467**	OPRM1 (opioid receptor mu 1)	Opioid receptor; modulation of pain and reward
**KH.C1.498**	CHAT (choline O-acetyltransferase)	Synthesis of the neurotransmitter acetylcholine
**KH.C1.696**	not found	
**KH.C1.731**	ECEL1 (endothelin converting enzyme like 1)	Neuropeptide processing and neuromuscular development
**KH.C1.935**	APOB (apolipoprotein B)	Lipid transport and metabolism
**KH.C1.1006**	not found	
**KH.C1.1067**	NCS1 (neuronal calcium sensor 1)	Calcium-dependent regulation of neurotransmitter release
**KH.C2.101**	SYT1 (synaptotagmin 1)	Calcium sensor for fast synchronous neurotransmitter release
**KH.C2.307**	DPYSL2 (dihydropyrimidinase like 2)	Regulation of axonal outgrowth and neuronal polarity
**KH.C2.862**	CORIN (corin, serine peptidase)	Serine protease; endocrine processing
**KH.C2.953**	not found	
**KH.C3.178**	SCGN (secretagogin, EF-hand calcium binding prote	Calcium binding and regulation of insulin/hormone release
**KH.C3.591**	CDHR5 (cadherin related family member 5)	Calcium-dependent cell-cell adhesion
**KH.C3.968**	CALM3 (calmodulin 3)	Primary calcium sensor; regulates diverse enzymatic activities
**KH.C3.969**	CALM3 (calmodulin 3)	Primary calcium sensor; regulates diverse enzymatic activities
**KH.C4.1**	ASH1L (ASH1 like histone lysine methyltransferase)	Histone methyltransferase; epigenetic gene regulation
**KH.C4.407**	KIF1B (kinesin family member 1B)	Anterograde transport of mitochondria and synaptic vesicles
**KH.C4.506**	HMCN2 (hemicentin 2)	Extracellular matrix structural component
**KH.C4.513**	not found	
**KH.C4.789**	MMP17 (matrix metallopeptidase 17)	Degradation of the extracellular matrix; tissue remodeling
**KH.C4.804**	GJC1 (gap junction protein gamma 1)	Gap junction communication; electrical coupling
**KH.C5.84**	PDS5B (PDS5 cohesin associated factor B)	Regulation of sister chromatid cohesion during the cell cycle and modulation of gene expression.
**KH.C5.117**	OPRM1 (opioid receptor mu 1)	G-protein coupled opioid receptor signaling
**KH.C5.230**	not found	
**KH.C5.232**	TIMP4 (TIMP metallopeptidase inhibitor 4)	Inhibition of metalloproteinases; ECM maintenance
**KH.C5.389**	TAC4 (tachykinin precursor 4)	Neuropeptide signaling (tachykinin family)
**KH.C5.481**	RPH3AL (rabphilin 3A like (without C2 domains))	Regulation of exocytosis in endocrine and neuronal cells
**KH.C6.149**	FAP (fibroblast activation protein alpha)	Cell surface proteolysis and tissue remodeling
**KH.C6.202**	PRSS35 (serine protease 35)	Serine protease activity; potential role in ovulation/ECM
**KH.C7.140**	OSBP2 (oxysterol binding protein 2)	Sterol transport and lipid sensing

**KH.C7.173**	SLC7A14 (solute carrier family 7 member 14)	Orphan transporter; likely lysosomal amino acid transport
**KH.C7.565**	VPS13D (vacuolar protein sorting 13 homolog D)	Regulation of mitochondrial and peroxisomal size
**KH.C8.400**	RAB3C (RAB3C, member RAS oncogene family)	Vesicle trafficking and exocytosis
**KH.C8.657**	GOLIM4 (golgi integral membrane protein 4)	Golgi-to-endosome retrograde transport
**KH.C8.710**	STC1 (stanniocalcin 1)	Regulation of calcium and phosphate homeostasis
**KH.C9.462**	SCN2A (sodium voltage-gated channel alpha subuni	Voltage-gated sodium channel for action potential firing
**KH.C9.763**	NMNAT2 (nicotinamide nucleotide adenylyltransfera	Essential for axonal maintenance and survival
**KH.C9.885**	AVPR1A (arginine vasopressin receptor 1A)	Vasopressin receptor; social behavior and homeostasis
**KH.C10.46**	RTN4 (reticulon 4)	Inhibition of neurite outgrowth and axonal regeneration
**KH.C10.528**	NCAM2 (neural cell adhesion molecule 2)	Cell adhesion and fasciculation of neurons
**KH.C12.22**	SH3PXD2A (SH3 and PX domains 2A)	Formation of podosomes/invadopodia; ECM degradation
**KH.C12.135**	LRRN2 (leucine rich repeat neuronal 2)	Neuronal cell adhesion
**KH.C12.218**	BCHE (butyrylcholinesterase); Ache (acetylcholinest	Termination of cholinergic signaling (acetylcholine breakdown)
**KH.C12.250**	not found	
**KH.C12.301**	FSTL5 (follistatin like 5)	Modulation of signaling pathways (e.g., TGF-beta)
**KH.L4.45**	not found	
**KH.L18.39**	MIB2 (MIB E3 ubiquitin protein ligase 2)	E3 ubiquitin ligase; Notch signaling regulation
**KH.L22.16**	IGFBP5 (insulin like growth factor binding protein 5)	Regulation of insulin-like growth factor (IGF) signaling
**KH.L22.28**	GABBR1 (gamma-aminobutyric acid type B receptor	Inhibitory neurotransmission via GABA-B receptors
**KH.L28.8**	FGF13 (fibroblast growth factor 13); Fgf11/12/13/14	Microtubule stabilization and sodium channel regulation
**KH.L56.3**	TTC28 (tetratricopeptide repeat domain 28)	Role in cytokinesis and cell cycle regulation
**KH.L68.2**	STMN2 (stathmin 2)	Microtubule destabilization; axonal growth
**KH.L108.41**	EIPR1 (EARP complex and GARP complex interacti	Intracellular protein trafficking (EARP/GARP complex)
**KH.L134.30**	GSTM3 (glutathione S-transferase mu 3)	Detoxification and protection against oxidative stress
**KH.L147.36**	GJA3 (gap junction protein alpha 3)	Gap junction formation and intercellular communication
**KH.L154.22**	ERBB2 (erb-b2 receptor tyrosine kinase 2)	Receptor tyrosine kinase signaling; cell proliferation
**KH.L170.66**	FASTKD2 (FAST kinase domains 2)	Mitochondrial ribosome assembly and energy metabolism
**KH.L172.15**	PLA2R1 (phospholipase A2 receptor 1)	Receptor for phospholipase A2; inflammatory signaling
**KH.S1051.1**	Gonadotropin-releasing hormone; GnRH	Regulation of reproductive development and behavior
**KH.S1104.4**	Gonadotropin-releasing hormone; GnRH	Regulation of reproductive development and behavior
**KH.S1313.1**	GTSE1 (G2 and S-phase expressed 1)	Regulation of cell cycle progression and p53 signaling

Supplemental Table S4. Genes predominantly expressed in anterior and posterior distal regions of the cerebral ganglion

**Gene ID**	**Protein (blast best hit)**	**Biological function**
**KH.C1.569**	HSPA8 (heat shock protein family A (Hsp70) member 8)	Molecular chaperone; protein folding and stress response
**KH.C2.491**	GOLGA4 (golgin A4)	Maintenance of Golgi structure and vesicle docking
**KH.C3.474**	MDGA2 (MAM domain containing glycosylphosphatidylinositol anchor	Regulation of synapse formation and cell adhesion
**KH.C4.437**	CAMK4 (calcium/calmodulin dependent protein kinase IV)	Calcium-dependent transcriptional regulation in neurons
**KH.C4.592**	KNTC1 (kinetochore associated 1)	Kinetochore assembly and chromosome segregation
**KH.C6.106**	PDE4DIP (phosphodiesterase 4D interacting protein)	Anchoring of phosphodiesterase; cAMP signaling
**KH.C7.399**	Calcitonin	Peptide hormone stimulates bone formation/inhibits bone resorption
**KH.C10.27**	Leu-Phe (LF) peptide family; ci-lf	novel peptide family in Ciona (Kawada et al., 2011)
**KH.C11.631**	ERICH1 (glutamate rich 1)	Putative signaling or structural role (glutamate rich)

Supplemental Table S5. Genes specifically expressed in the medulla of the cerebral ganglion

Gene ID	Protein (blast best hit)	Biological function
**KH.C1.342**	CACNA2D1 (voltage-gated calcium channel auxiliary subunit alpha2del	Regulation of calcium channel expression and activation
**KH.C3.99**	SPIRE1 (spire type actin nucleation factor 1); Pem5 (Posterior end mar	Actin nucleation and vesicle transport
**KH.C6.289**	ADAM7 (ADAM metallopeptidase domain 7)	Cell-cell interaction and protein ectodomain shedding
**KH.C8.717**	CPLX2 (complexin 2)	Regulation of synaptic vesicle fusion and neurotransmission
**KH.C8.892**	TUBA3D (tubulin alpha 3d)	Structural component of microtubules
**KH.C9.147**	USH2A (usherin)	Structural component of basement membranes
**KH.C9.150**	DCLK2 (doublecortin-like and CAM kinase-like 2)	Microtubule polymerization and neuronal migration
**KH.C10.335**	CALM3 (calmodulin 3)	Intracellular calcium signal transduction
**KH.C10.493**	ZNF704 (zinc finger protein 704)	Transcriptional regulation via zinc finger binding
**KH.C11.168**	RIN1 (Ras and Rab interactor 1)	Ras effector; regulation of endocytosis and actin
**KH.C14.470**	SYNE2 (spectrin repeat containing nuclear envelope protein 2)	Linking the nucleus to the cytoskeleton
**KH.L7.5**	RAB39B (RAB39B, member RAS oncogene family)	Vesicular trafficking and neuronal maturation
**KH.L20.63**	not found	
**KH.L55.2**	CALM3 (calmodulin 3)	Calcium-dependent enzymatic regulation
**KH.L113.1**	RUFY2 (RUN and FYVE domain containing 2)	Endosomal tethering and vesicle fusion
**KH.L116.85**	TUBB4B (tubulin beta 4B class IVb); Tubb (tubulin beta)	Primary structural unit of microtubules
**KH.S605.3**	CRYBB2 (crystallin beta B2); Beta/gamma crystallin	Structural protein; also has non-refractive functions
**KH.S854.1**	BRI3 (brain protein I3)	Regulation of neuronal signaling and cell death
**KH.S1622.1**	MYO3B (myosin IIIB)	Actin-based motor protein; ciliary/microvilli maintenance
